# Randomised study of adjuvant chemotherapy for completely resected p-stage I–IIIA non-small cell lung cancer

**DOI:** 10.1038/sj.bjc.6603336

**Published:** 2006-09-12

**Authors:** K Nakagawa, H Tada, A Akashi, T Yasumitsu, K Iuchi, T Taki, K Kodama, The Osaka Lung Cancer Study Group, Japan

**Affiliations:** 1 Department of Thoracic Surgery, Osaka Prefectural Medical Center for Respiratory and Allergic Diseases, 3-7-1 Habikino, Habikino 583-8588, Japan; 2 Department of Pulmonary Surgery, Osaka City General Hospital, Osaka 534-0021, Japan; 3 Department of Thoracic Surgery, Takarazuka Municipal Hospital, Takarazuka 665-0827, Japan; 4 Department of Surgery, National Kinki Central Hospital for Chest Diseases, Sakai 591-8555, Japan; 5 Department of Thoracic Surgery, Kitano Hospital, The Tazuke Kofukai Medical Research Institute, Osaka 530-8480, Japan; 6 Department of Thoracic Surgery, Osaka Medical Center for Cancer and Cardiovascular Diseases, Osaka 537-8511, Japan

**Keywords:** adjuvant chemotherapy, complete resection, non-small cell lung cancer, DNA ploidy pattern, randomised controlled trial, UFT

## Abstract

We evaluated the therapeutic usefulness of adjuvant chemotherapy in patients with completely resected non-small cell lung cancer (NSCLC). We also examined the relation between DNA ploidy pattern and the response to chemotherapy. A total of 267 patients with NSCLC (pathologically documented stage I, II, or IIIA) underwent complete resection, and DNA ploidy pattern was analysed. Patients with stage I disease (*n*=172) were randomly assigned to receive surgery alone (group A) or surgery followed by adjuvant chemotherapy (UFT (oral anti-cancer drug, a combination of Uracil and Tegaful) 400 mg day^−1^ for 1 year after surgery; group B). Stage II or IIIA disease patients (*n*=95) were randomly assigned to surgery alone (group C) or surgery followed by chemotherapy (two 28-day courses of cisplatin 80 mg m^−2^ on day 1 plus vindesine 3 mg m^−2^ on days 1 and 8, followed by UFT 400 mg day^−1^ for at least 1 year; group D). Eight-year overall survival rate in patients with stage I disease was 74.2% (95% confidence interval (CI): 64.4–84.0%) in group B and 57.6% (95% CI: 46.4–68.8%) in group A (*P*=0.045 by log-rank test). In patients with stage II and IIIA disease, no difference was found between groups C and D. Analysis according to DNA ploidy pattern revealed no difference between the groups. Postoperative chemotherapy with UFT was suggested to be useful in patients with completely resected stage I NSCLC. No difference was seen in relation to DNA pattern in any treatment group.

A meta-analysis of postoperative chemotherapy in non-small cell lung cancer (NSCLC) reported by the British Medical Council in 1995 found that adjuvant chemotherapy did not adequately improve outcome in this condition (Non-small Cell Lung Cancer Collaborative Group, 1995). Despite a number of trials since, the value of postoperative chemotherapy for NSCLC remains controversial (Wada *et al*, 1996; Endo *et al*, 2003; Scagliotti *et al*, 2003). Beginning around 1990, considerable attention has been focused on DNA ploidy pattern as a possible new prognostic factor, with tumours showing aneuploidy, associated with a poor prognosis, reported to show a better response to chemotherapy than those showing diploidy (Granone *et al*, 1993; Salvati *et al*, 1994; Kim *et al*, 1996). However, these previous studies were based on retrospective data.

Here, we investigated the usefulness of postoperative adjuvant chemotherapy for the management of NSCLC patients prospectively assigned to treatment on the basis of DNA ploidy.

## PATIENTS AND METHODS

### Eligibility criteria

Eligibility criteria included an untreated primary lung cancer; histologically confirmed diagnosis of squamous cell carcinoma, adenocarcinoma, or large cell carcinoma; pathologically documented stage I, II, or IIIA disease; diploidy or aneuploidy on analysis of nuclear DNA of the primary tumour; age 75 years or younger in patients with stage I disease or 70 years or younger in those with stage II or IIIA disease; Eastern Cooperative Oncology Group (ECOG) performance status of 0, 1, or 2; and adequate organ function as defined by a leucocyte count of at least 4000 mm^−3^, platelet count of at least 100 000 mm^−3^, serum haemoglobin level of at least 10 g dl^−1^, serum aspartate aminotransferase (AST) level of not more than 100 U, alanine aminotransferase (ALT) level of at most 100 U, albumin/globulin ratio of at least 1.0, serum creatinine level of less than 1.5 mg dl^−1^, and serum urea nitrogen level of not more than 25 mg dl^−1^. Further, patients with a serious concurrent condition were also excluded. All tumours were resected by pulmonary resection consisting of at least lobectomy and systematic hilar/mediastinal lymph node dissection. Cases of complete resection were defined as those without macroscopic residual tumour or microscopic positive margins. The study was reviewed and approved by the institutional review boards of each participating centre, and written informed consent was obtained from all patients.

Because stage I disease differs considerably from stage II and IIIA disease, assignment of similar treatments would have negatively affected outcome. Patients with stage II or IIIA disease were therefore assigned to receive different treatment from those with stage I disease.

### Measurement of DNA ploidy

Samples were harvested and frozen immediately after tumour excision. Nuclear DNA content was measured by flow cytometry and evaluated by an independent flow cytometry evaluation committee who were blinded to patient data.

### Treatment schedule

Patients were grouped according to stage as follows. For stage I patients, Group A (control) received no adjuvant chemotherapy but was followed after surgery, whereas Group B received single daily oral administration of UFT (oral anti-cancer drug, a combination of Uracil and Tegaful) at 400 mg day^−1^ for at least 1 year starting 3–6 weeks after surgery.

For stages II and IIIA, Group C (control) received no adjuvant chemotherapy but was followed after surgery, whereas Group D was given two 28-day courses of chemotherapy with cisplatin (80 mg m^−2^) on day 1 and vindesine (3 mg m^−2^) on days 1 and 8, starting 3–6 weeks after surgery, followed by single daily oral administration of UFT at 400 mg day^−1^ for at least 1 year.

Adverse effects of chemotherapy were evaluated using the National Cancer Institute Common Toxicity Criteria (version 2.0, Jan 30, 1998) and handled by appropriate treatment, discontinuation of UFT, or both. In Group D, chemotherapy after the administration of vindesine on day 8 of the first course was continued only after confirmation that white blood cell count was greater than 3000. Other anticancer drugs, immunomodulators, and radiotherapy were not used unless recurrence was confirmed.

### Treatment assignment

Patients were stratified on the basis of pathological stage, histologic type, and ploidy pattern, and then randomly assigned to groups. Randomisation was performed centrally, with assignment for pathological T and N stage balanced using the minimisation method with probability and the method of Zelen (Yonekura *et al*, 1999; Tanaka *et al*, 2002).

### Statistical analysis

The primary end point was overall survival, defined as the time from surgery until death from any cause. The secondary end point was disease-free survival, defined as the time from surgery until relapse or death from any cause, whichever occurred first.

Survival curves were calculated by the Kaplan–Meier method (Kaplan and Meier, 1958), and statistical significance of differences between groups was compared with the log-rank test (Peto *et al*, 1977). *P*-values of less than 0.05 were considered to indicate significance. Multivariable analysis to estimate the simultaneous effects of prognostic factors on survival was carried out with the Cox proportional-hazards model. Categorical variables were compared using the *χ*^2^ test.

Target numbers of patients were calculated as follows. Based on previous studies (Kuwahara *et al*, 1989; The Study Group of Adjuvant Chemotherapy for Lung Cancer, 1995; Wada *et al*, 1996), the assumed 5-year survival in the control group was 75% in stage I patients and 40% in stage II or III patients. The expected survival improvement was 15% in stage I patients and 25% in stage II or III patients.

On the basis of Freedman's sample size table (Freedman, 1982), the sample size required to detect a significant difference between surgery alone and surgery plus chemotherapy at a power of 0.8 and a 5% level of significance was 169 patients with stage I disease and 102 with stage II, or III disease.

One goal of this study was to compare survival in patients with aneuploid tumours who received chemotherapy after surgery with that in patients who received surgery alone. Given an aneuploid to diploid tumour ratio of 8:2 (Yamaoka *et al*, 1991), 80% of stage I, II, and III cases were estimated to be aneuploid. The required number of cases was therefore estimated to be 212 cases of stage I disease and 128 cases of stage II or III disease. Allowing for 8% ineligibility and loss to follow-up, the target number was set at 230 patients with stage I and 140 with stage II or III disease.

All statistical analyses were carried out using the SAS software package ver. 7 (SAS Institute Inc, Cary, NC, USA).

## RESULTS

A total of 287 patients were enrolled at 15 centres from April 1992 to March 1994. At this time, a number of new induction chemotherapy protocols for early stage lung cancer were introduced in Japan, hampering the further accrual of patients, and enrollment was therefore stopped in March 1994. This report evaluates cases followed until the end of November 2001.

Of the 287 patients enrolled, 20 were excluded because they did not meet the entry criteria, namely conditions other than cancer (inflammatory or benign tumours) or non-curative resection. Median follow-up time of the 267 patients studied was 7.4 years.

### Clinical characteristics

Of 172 patients with stage I disease, 87 were assigned to group A and 85 to group B, with no significant difference between them in sex, mean age, performance status, T stage, histologic type, or tumour DNA pattern.

Of 95 patients with stage II (*n*=33) or IIIA (*n*=62) disease, 48 were assigned to group C and 47 to group D, with no significant difference between them in sex, mean age, performance status, T stage, N stage, pathologically determined disease stage, histologic type, or DNA pattern (Table 1).

### Treatment rate

A 100% treatment rate was defined as at least 1 year of continuous treatment with 400 mg of UFT daily. From this, individual rates were calculated as (number of UFT administration days)/365 × (daily UFT dosage)/400 × 100. Mean treatment rates in groups B and D were estimated to be 76.7 and 48.6%, respectively. In group D, 39 patients were given one or more courses of cisplatin and vindesine (one course in three patients, two in 36). Treatment compliance was 83.0%. Treatment was not given to eight patients owing to patient refusal in two, postoperative complications in two, and poor general condition in four.

### Survival rate

**Figure 1:**
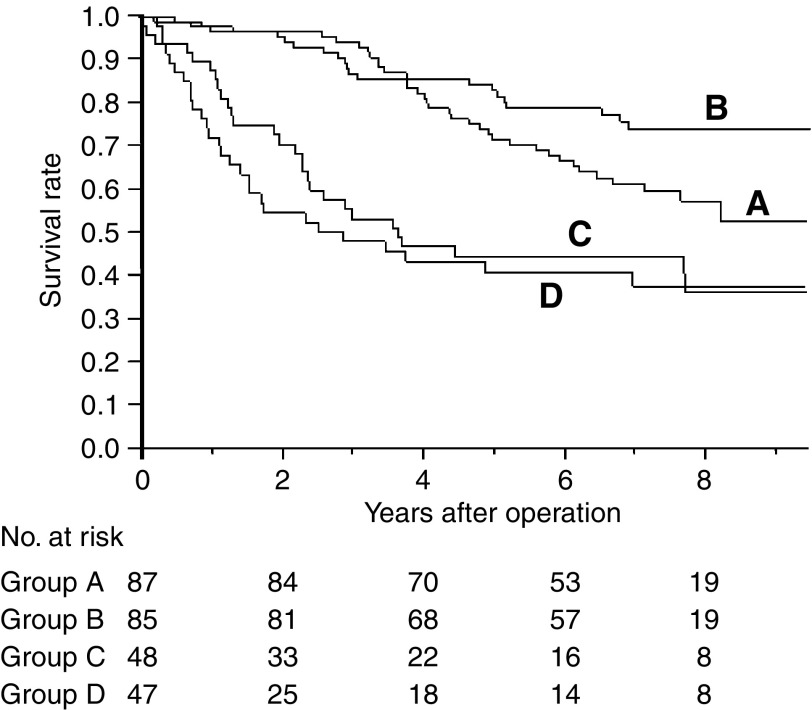
Overall survival of stage I control and UFT group patients. Eight-year survival rate was 57.6% for the control group **A** (*n*=87) and 74.2% for the UFT group **B** (*n*=85). A significant difference in survival curves for these groups is seen (*P*=0.045 by log-rank test).

Eight-year overall survival rate in stage I patients was 57.6% (95% confidence interval (CI): 46.4–68.8%) in group A (control) and 74.2% (95% CI: 64.4–84.0%) in group B, with a significant difference seen between the two survival curves (*P*=0.045; Figure 1). Respective rates in Groups C and D were 36.8% (95% CI: 21.3–52.3%) and 38.0% (95% CI: 23.5–52.5%), with no significant difference between these two overall survival curves (*P*=0.52). Moreover, no significant difference was seen in 8-year disease-free survival rate between groups A and B or C and D.

### Subgroup analyses

When subgrouped by histology, the adenocarcinoma subgroup showed no significant difference in overall survival curves between groups A and B (*P*=0.065); or between patients in groups A and B with diploid tumours (*P*=0.078) or aneuploid tumours (*P*=0.16) (Table 2). Moreover, overall survival curves in this adenocarcinoma subgroup did not differ significantly between groups C and D (group C, *n*=29, 8-year survival rate 41.3%, 95% CI: 21.9–60.7%; group D, *n*=27, 8-year survival rate 40.0%, 95% CI: 19.9–60.0%; *P*=0.98).

When subgrouped by DNA pattern, no significant difference in overall survival curve was seen in diploid tumour patients in groups C and D (group C, *n*=10, 8-year survival rate 30.0%, 95% CI: 1.6–58.4%; group D, *n*=8, 8-year survival rate 37.5%, 95% CI: 4.0–71.0%; *P*=0.92); or between aneuploid patients in groups C and D (group C, *n*=38, 8-year survival rate 36.8%, 95% CI: 17.8–55.8%; group D, *n*=39, 8-year survival rate 38.1%, 95% CI: 21.8–54.4%; *P*=0.44).

### Multivariate analysis

Multivariate analysis indicated that UFT treatment was a significant predictor of outcome in stage I patients, following age and sex (Table 3).

### Adverse effects

Grade 3 anorexia occurred in one patient in group B. The incidence of grade 3 and 4 toxicity was only 1.2% in group B. In group D, grade 3 or 4 leucopenia was seen in seven patients (14.6%), anorexia in three patients (6.3%), nausea/vomiting in two patients (4.2%), and hair loss in one patient (2.1%) (Table 4). No episode lasted more than 1 month.

There were no lethal adverse effects in either chemotherapy group.

### Mortality

Twenty-six of 87 patients (29.9%) in group A and 14 of 85 (16.5%) in group B died of their tumours, with this difference being significant (*P*=0.037). Nine patients in group A and six in group B died of causes other than cancer, with this difference not significant (*P*=0.45).

The rate of mortality from cancer was 52.1% in group C and 42.6% in group D, with this difference not significant (*P*=0.35). The rate of mortality from other causes was 6.3% in group C and 17.0% in group D, again without significance (*P*=0.10).

## DISCUSSION

UFT is an oral fluorinated dihydropyrimidine preparation which combines tegafur, a prodrug of 5-fluorouracil, with uracil, an inhibitor of dihydropyrimidine dehydrogenase, the enzyme which catalyses the metabolism of 5-fluorouracil. One reason for the effectiveness of postoperative adjuvant chemotherapy with UFT in patients with completely resected stage I lung cancer is the action of tegafur. The metabolism of tegafur results in prolongation of active levels of 5-fluorouracil, and its metabolites (GHB and GBL) promote angiogenesis (Yonekura *et al*, 1999; Basaki *et al*, 2001; Tanaka *et al*, 2002). Meta-analysis of several randomised controlled studies has confirmed that UFT is therapeutically useful. The Japan Lung Cancer Research Group (JLCRG) performed Phase III randomised controlled studies of postoperative adjuvant chemotherapy with UFT in patients with stage I adenocarcinoma of the lung, and showed significant improval in survival, with a hazard ratio of 0.71 (95% CI: 0.52–0.98) as compared with surgery alone in this subgroup (*P*=0.04) (Kato *et al*, 2004).

Our study enrolled a wider range of patients than the JLCRG study, including not only adenocarcinoma and completely resected stage I NSCLC, but also stage II and IIIA NSCLC. Survival in patients with stage I disease was significantly better in group B than in group A (*P*=0.045). On analysis by histologic tumour type, survival in patients with adenocarcinoma was slightly but not significantly better in group B (*P*=0.065). In contrast, survival in patients with diploid tumours did not differ between group A and B, probably because of the low patient numbers, which were insufficient for statistical analysis (*n*=18 and 17, respectively). The number of patients with diploid tumour was small as compared with that of patients with aneuploid tumour, and was insufficient for statistical analysis in the present study.

In stage II and III adenocarcinoma, in contrast, no difference in survival was seen either overall, or by ploidy. These results therefore suggest that UFT is effective for the management of stage I lung cancer, consistent with the findings of the WJSG 2nd study and the JLCRG study (Kato *et al*, 2004).

Early studies of DNA ploidy (Granone *et al*, 1993; Salvati *et al*, 1994; Kim *et al*, 1996) reported that aneuploidy is an independent predictor of poor outcome. In contrast, more recent investigations (Fujino *et al*, 1996; Bellotti *et al*, 1997; Reinmuth *et al*, 2000; Pelletier *et al*, 2001) have questioned the value of ploidy as a prognostic factor. Statistical analysis of the prognostic implications of diploidy was precluded in the present study owing to the low number of patients with diploid tumours (stage I, *n*=35; stage II and III, *n*=18).

With regard to the efficacy of the present therapeutic regimen, a meta-analysis of the usefulness of postoperative cisplatin-based adjuvant chemotherapy in NSCLC found good efficacy in stage II and III patients receiving cisplatin-based (320 mg m^−2^ or more) chemotherapy with vinorelbine (Pignon *et al*, 2006). In contrast, the dose of cisplatin in the present study was as low as 160 mg m^−2^ and vindesine was used as a combination drug. These differences are likely associated with the insufficient efficacy seen here. Further, the response rate to UFT in unresectable NSCLC has been reported as only 8% (Keicho *et al*, 1986), and usefulness of postoperative adjuvant chemotherapy has been described for relatively early-stage NSCLC only. The possibility therefore exists that UFT may have insufficient efficacy in stage II/III disease with high malignancy.

In conclusion, although the relation between DNA ploidy pattern and the response to postoperative adjuvant chemotherapy remains unclear, our results suggest that postoperative adjuvant chemotherapy with UFT improves survival and is therapeutically useful in patients with completely resected stage I NSCLC.

## Figures and Tables

**Table 1:** Patient characteristics

	**A**	**B**	**Total**	**C**	**D**	**Total**
No. of eligible patients	87	85	172	48	47	95
*Sex*						
Male	49	49	98	35	35	70
Female	38	36	74	13	12	25
						
*Age*						
(Ave.)	60.9	60.2	60.6	59.3	60.5	59.9
						
*PS*						
0	66	66	132	36	35	71
1	19	19	38	12	10	22
2	2	0	2	0	2	2
						
*pT*						
1	41	45	86	12	13	25
2	46	40	86	24	24	48
3				12	10	22
						
*pN*						
0				6	1	7
1				18	19	37
2				24	27	51
						
*Stage*						
I	87	85	172			
II				16	17	33
IIIA				32	30	62
						
*Histology*						
Adenocarcinoma	67	68	135	29	27	56
Squamous cell carcinoma	17	15	32	17	17	34
Large cell carcinoma	3	2	5	2	3	5
						
*DNA pattern*						
Diploidy	18	17	35	10	8	18
Aneuploidy	69	68	137	38	39	77

Abbreviations: PS, performance status; pT, pathological tumour stage; pN, pathological lymph node stage.

**Table 2:** Overall survival in the control and UFT groups

	**No. of cases**		**8-year survival rate (%)**		
	**A**	**B**	**A**	**B**	***P*-value**
*Sex*					
Female	38	36	74.7	78.0	0.850
Male	49	49	43.6	71.6	0.013
					
*Age* (years)					
<60	38	37	74.5	84.8	0.171
⩾60	49	48	45.9	65.4	0.153
					
*PS*					
0	66	66	62.0	78.3	0.055
1, 2	21	19	43.4	62.2	0.460
					
*pT*					
T1	41	44	56.4	87.7	0.014
T2	46	40	59.4	58.5	0.763
					
*Histology*					
Adeno carcinoma	67	68	60.2	75.6	0.065
Non-adeno carcinoma	20	17	51.7	69.3	0.503
					
*DNA pattern*					
Diploidy	18	17	53.5	86.7	0.078
Aneuploidy	69	68	59.0	71.3	0.158

Abbreviations: PS, performance status; pT, pathological tumour stage.

**Table 3:** Multivariate analysis of outcomes

**Factor**	**Hazard ratio**	**95% CI**	***P*-value**
*Sex*			
Female	1		
Male	1.95	1.11–3.60	0.019
			
*Age* (years)			
<60	1		
⩾60	2.24	1.26–4.20	0.0053
			
*Group*			
Control	1		
UFT	0.57	0.32–0.97	0.039

**Table 4:** Toxicity

	**UFT**						**CDDP+VDS+UFT**				
	**Grade**						**Grade**				
**Toxicity (*n*=85)**	**1**	**2**	**3**	**4**	**Frequency of G3 or G4 (%)**	**Toxicity (*n*=47)**	**1**	**2**	**3**	**4**	**Frequency of G3 or G4 (%)**
Leucopenia	8	2	0	0		Leucopenia	8	11	5	2	14.6
Thrombocytopenia	2	0	0	0		Thrombocytopenia	3	2	0	0	
Anaemia	1	0	0	0		Anaemia	8	6	0	0	
AST	6	0	0	0		GOT	6	3	0	0	
ALT	5	2	0	0		GPT	9	4	0	0	
Anorexia	10	8	1	0	1.2	BUN	5	0	0	0	
Nausea/vomiting	8	1	0	0		Creatinine	2	0	0	0	
Diarrhoea	3	1	0	0		Anorexia	10	14	3	0	6.3
Stomatitis	4	0	0	0		Nausea/vomiting	12	4	2	0	4.2
Pigmentation	6	0	0	0		Diarrhoea	4	1	0	0	
Alopecia	0	1	0	0		Stomatitis	4	0	0	0	
						Alopecia	7	7	1	0	2.1

Abbreviations: BUN, blood urea nitrogen; CDDP, cisplatin; VDS, vindesine sulfate.

## **Appendix A**

- Osaka University, Faculty of Medicine
- Osaka Medical Center for Cancer and Cardiovascular Diseases
- Osaka Prefectural Medical Center for Respiratory and Allergic Diseases
- Kansai Medical University
- Kansai Electric Power Hospital
- Kinki University School of Medicine
- Toneyama National Hospital
- National Kinki Central Hospital for Chest Diseases
- Sumitomo Hospital
- Takarazuka Municipal Hospital
- Kitano Hospital The Tazuke Kofukai Medical Research Institute
- Osaka City General Hospital
- Hyogo College of Medicine
